# Computational fluid dynamics assessment of altered hemodynamics in the Circle of Willis during acute ischemic stroke and the impact of cerebral collateral development

**DOI:** 10.1007/s10237-026-02060-y

**Published:** 2026-03-27

**Authors:** Cody Kubicki, Scott Simon, Keefe B. Manning

**Affiliations:** 1 Department of Biomedical Engineering, The Pennsylvania State University, University Park, PA USA; 2 Department of Neurosurgery, Penn State College of Medicine, Hershey, PA USA; 3 Department of Surgery, Penn State College of Medicine, Hershey, PA USA

**Keywords:** Acute ischemic stroke, Computational fluid dynamics, Cerebral hemodynamics, Leptomeningeal collateral circulation, Thrombolytic therapy, Endovascular thrombectomy

## Abstract

Cerebral collateral assessment has become a common metric for treatment planning in acute ischemic stroke patients due to clinical evidence that well-developed collateral networks are correlated with favorable patient outcomes for reperfusion therapies, such as intravenous thrombolytics and mechanical thrombectomy. However, the mechanisms driving these outcome disparities are not well clarified. In the present study, a computational model is used to help clarify these mechanisms by assessing the Circle of Willis hemodynamics during middle cerebral artery occlusion with different levels of collateral development present. The results showed that middle cerebral artery occlusion causes up to a 30% increase in systemic mean arterial pressure, but the increase is less severe in cases with better collateralization, and cases with well-developed collaterals had up to a 66% lower pressure drop across the clot compared to the cases with poor collateral development. The ipsilateral collateral flow increased up to 20-fold following occlusion, which elevated blood flow and mixing distal to the occlusion. These results indicate that cerebral collaterals serve multiple functions that are important to consider in stroke cases. First, collaterals compensate for part of the lost blood flow to the affected brain region by permitting retrograde flow toward the distal end of the occluded vessel. Second, collaterals reduce the pressure forces on the clot, which can improve the susceptibility to reperfusion therapies. Overall, this study shows that we can leverage our unique computational model to better understand the importance of cerebral collateral circulation during stroke and the influence of collaterals on therapeutic outcomes.

## Introduction

Acute ischemic stroke (AIS) is one of the leading global causes of morbidity and mortality (Martin et al. 2024; Feigin et al. 2025). With an aging population, the health and financial burden of AIS are increasing annually (Feigin et al. 2024). The severity and prevalence of this disease underscore the need to develop improved treatment strategies, leading to more favorable functional outcomes. AIS is a severe disease, and effective treatment is extremely time sensitive (Tsivgoulis et al. 2020; Herm et al. 2021; Uniken Venema et al. 2022b). Particularly in cases of large vessel occlusions (LVO) in proximal vessels, such as the M1 segment of the middle cerebral artery (M1-MCA) and internal carotid artery (ICA), the occluded vessel must be rapidly recanalized to restore perfusion to the downstream brain tissue and limit the brain infarct volume (Munsch et al. 2024). Prior to recanalization, the distal brain tissue that is normally supplied by the occluded vessel relies solely on blood supplied by the leptomeningeal collateral network (LCN). The LCN is a vast network of connected penetrating arterioles, pial arteries, and microvascular anastomoses. The pial arteries of the LCN connect the distal ends of the primary vessels in the Circle of Willis (CoW) and run along the surface of the cerebral hemisphere. Pial arteries stemming from different large proximal vessels are connected by microvascular anastomoses both along the cortical surface and within the brain parenchyma to complete the collateral network. These smaller anastomoses serve as vascular connections to permit blood exchange between brain territories that are perfused by different large vessels in the CoW. Under normal physiological flow conditions, the pressure gradient through the LCN vessels is relatively low, resulting in slow sustained bidirectional antegrade flow that progresses away from the CoW and toward the perforating arterioles that branch off the pial arteries into the brain parenchyma to perfuse local tissue (Brozici et al. 2003; Shuaib et al. 2011). However, LVO can substantially change the pressure gradient across these collateral vessels, resulting in acute collateral recruitment. Collateral recruitment occurs because the occlusive clot has an extremely high hemodynamic resistance, which causes the pressure distal to the occlusion to fall drastically and creates an altered pressure gradient through the collateral vessels. This pressure gradient increases unidirectional retrograde flow through the collaterals from the remaining patent arteries on the ipsilateral side to the distal region of the occluded vessel. The retrograde blood flow from one arterial brain territory to another helps to facilitate perfusion of the region downstream of the occlusion and minimize the extent of brain tissue damage (Romero et al. 2009; Beard et al. 2015; Kim et al. 2021; Uniken Venema et al. 2022a). To further increase flow through the collateral network, arterial resistance is reduced through cell-mediated vessel dilation in the pial and microvascular collateral arteries. This acute phase collateral recruitment and vessel dilation due to pressure changes can occur in as little as 12 s and result in as much as a sixfold increase in collateral vessel size and nearly a ninefold increase in collateral flow rate, as shown in animal LVO models (Coyle 1984; Morita et al. 1997; Litman et al. 2025). Together, these physiological processes are intended to temporarily compensate for the loss of blood flow through the occluded vessel and maintain perfusion of the affected brain tissue.

The LCN anatomy can vary greatly between individuals (Vander Eecken 1954). Factors that have been associated with poor collateral development include age, sex, chronic hypertension, and diabetes (Li et al. 2024). There is an abundance of clinical evidence that the level of collateral development in an individual, as assessed by arterial inflow to ischemic brain, tissue perfusion, and venous outflow quantified in computed tomography (CT) perfusion scans, is a strong predictor of clinical outcomes for AIS patients (Lima et al. 2010; Fanou et al. 2015; Jiang et al. 2019; Lin et al. 2021; Saber and Liebeskind 2021). The associated studies have shown that poorly developed collaterals with low compensatory capacity are associated with increased brain tissue infarct volume growth rates and poorer clinical outcomes. As a result, collateral development level has recently become a clinical indicator for reperfusion therapy. Collateral assessment and scoring have become popular in large part due to the increased availability and use of advanced neuroimaging techniques, such as CT angiography (CTA), CT perfusion, and magnetic resonance (MR) perfusion, in AIS cases that permit small vessel visualization (Tariq and Khatri 2008). For some neurosurgical centers, this has shifted the treatment paradigm from a time-based to tissue-based assessment rooted in the idea of the “collateral clock” where increased collateralization extends the effective treatment window because the infarct volume grows at a slower rate, and there remains a greater volume of recoverable tissue within the ischemic penumbra when the collateral network can compensate for lost blood flow (Maguida and Shuaib 2023). Therefore, the collateral circulation has become a critical consideration for clinicians when evaluating patient eligibility for acute reperfusion therapies, such as endovascular thrombectomy (EVT).

In addition to its role of maintaining blood perfusion to ischemic brain territories during AIS, there is evidence that the LCN plays an important role in other hemodynamic and biomechanical mechanisms that impact the success of reperfusion therapies because studies have shown patients presenting with favorable cerebral collateral profiles are more likely to achieve vessel recanalization following thrombolytic administration (Wufuer et al. 2017; Seners et al. 2019; Faizy et al. 2022). Altered pressure forces experienced by the clot and changes to the surrounding blood flow on the distal and proximal ends mediated by the LCN are hypothesized to factor into recanalization success of intravenous thrombolytic therapy (IVT) and EVT. Poor collateral flow is associated with occlusive thrombus extension in LVO cases in the distal ICA or proximal MCA, which occurs due to distal flow stasis. Patients that experienced this thrombus extension effect had worse outcomes following IVT (Zhang et al. 2018). Collaterals are hypothesized to play a secondary role in clot lysis and vessel recanalization by introducing more retrograde flow and transport of thrombolytic proteins to the distal surface of the clot from the vessels downstream of the occlusion site (Liebeskind et al. 2014; Uniken Venema et al. 2022a). However, these hypotheses have yet to be extensively tested and proven in either an experimental or computational setting. Leaving a knowledge gap surrounding the physical mechanisms driving the observed clinical correlations.

To date, very few computational studies have focused on elucidating the effect of collateral circulation on hemodynamics during AIS (Phan et al. 2014; Kennedy McConnell and Payne 2017; Padmos et al. 2021; Otani et al. 2023) despite the large amount of clinical evidence indicating its importance. These previous studies have largely used one-dimensional steady flow models, which do not capture the true three-dimensional anatomy and pulsatile flow conditions experienced in vivo (Phan et al. 2014; Kennedy McConnell and Payne 2017; Padmos et al. 2021; Otani et al. 2023). One previous model that did include a three-dimensional anatomical model used over-simplified straight pipes to simulate cerebral vessel geometry (Phan et al. 2014). In the present study, we develop a three-dimensional computational model that can recapitulate the hemodynamics within the CoW of individuals in the elderly demographic (> 60 years old) both before and during AIS. The goal of our study is to leverage this computational model to observe hemodynamic changes in the major cerebral vessels and within simulated cerebral collateral networks following LVO in the M1-MCA. These simulations are repeated in multiple cerebral vessel anatomies representing varying degrees of pre-stroke collateral development to better understand the impact that cerebral collateral networks have on blood flow, convective transport to the distal end of the clot, and the pressure forces experienced by the clot following vessel occlusion. Collectively, these are thought to be important mechanisms for predicting recanalization success and patient outcomes of AIS patients.

## Methods and models

### Anatomical model

**Fig. 1:**
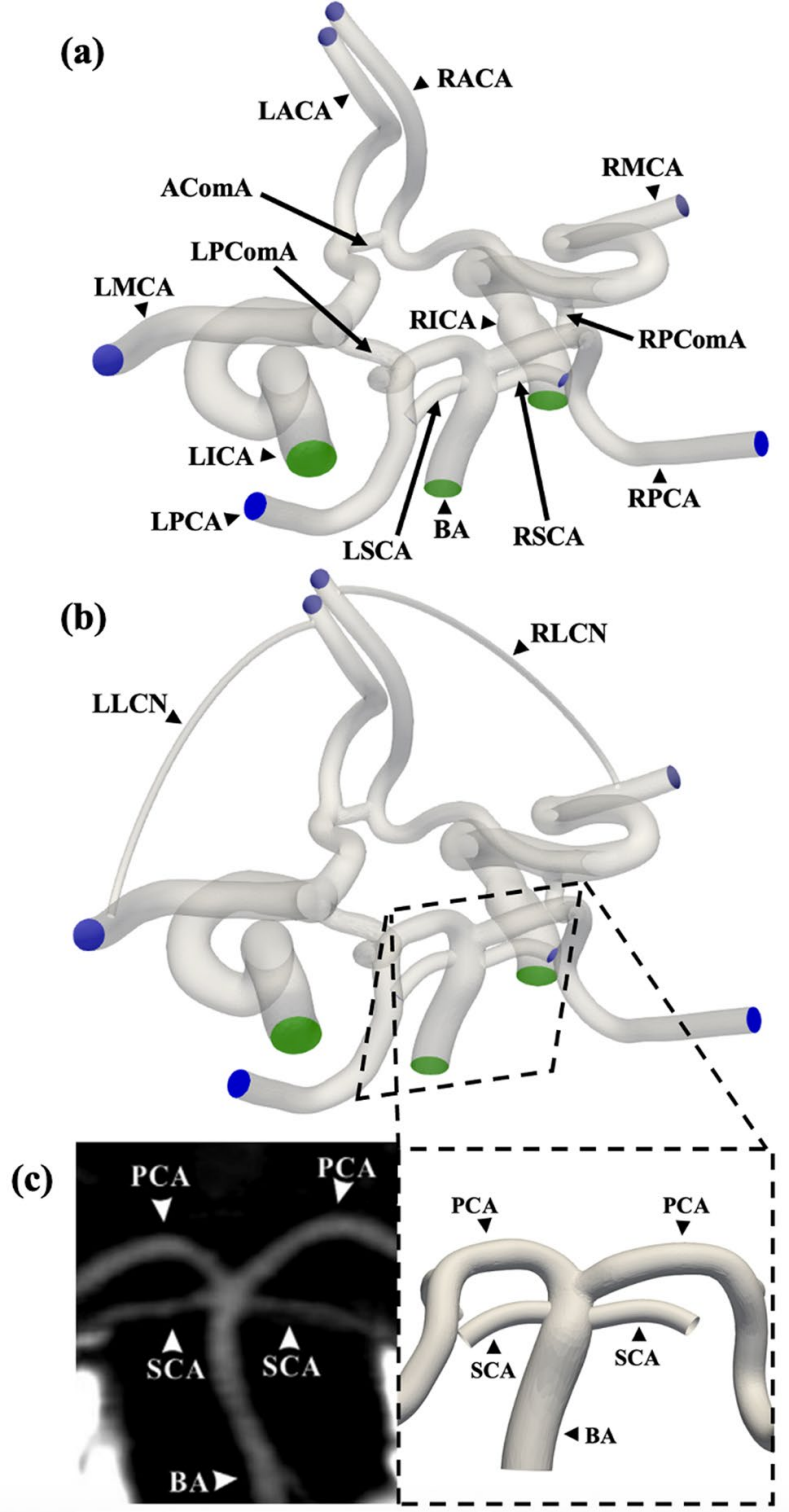
Renderings of the anatomical geometry of the Circle of Willis used in the simulations (**a**) without and (**b**) with the simulated LCN from the distal ACA to MCA with the major vessels labeled (green shaded regions highlight the inlet vessel boundaries and blue shaded regions are the outlet vessel boundaries), and (**c**) detailed images of the isolated proximal posterior circulation comparing a representative CTA scan of a typical SCA configuration with bilateral origins from the BA (left figure from (Krzyżewski et al. 2014)) to the computational mesh

A reconstructed healthy adult CoW created from segmented and reconstructed CTA data was used as the baseline anatomical model for this study (Figueroa 2020). This model represents a typical complete CoW with all the major cerebral vessels including the basilar artery (BA), left and right internal carotid arteries (ICA), left and right middle cerebral arteries (MCA), left and right posterior cerebral arteries (PCA), left and right anterior cerebral arteries (ACA), as well as the posterior (PComA) and anterior (AComA) communicating arteries. Alpers et al. (1959) found that 52% of adults with no evidence of vascular pathology presented with a complete CoW, like the baseline anatomical model used in this study. The baseline model was modified using Blender 3.6.1 (Blender Foundation; Amsterdam, Netherlands) to include two additional branches from the terminus of the basilar artery to represent the left and right superior cerebellar arteries (SCA) and an anastomosis between the distal ACA and the distal MCA on both sides. Images of the modified reconstructions used to generate the computational meshes are shown in Fig. 1a, b.

The smaller SCAs branching from the BA had to be added manually because they are less commonly found segmented in CTA data. However, their inclusion in the model was necessary to account for posterior circulation flow loss from the proximal BA, where the clinical flow data were measured, to the left and right PCA branches due to flow diversion to the SCAs. Thus, SCA inclusion allowed the model to maintain continuity through the system while matching clinical flow data through the primary CoW vessels. Although the three-dimensional vessel geometries in the model were not directly derived from patient CTA data, they were designed to match the most common configuration (symmetric bilateral branching from the BA) and average SCA vessel internal diameter from the BA origin (1.48 mm) (Krzyżewski et al. 2014), as shown in Fig. 1c.

**Fig. 2:**
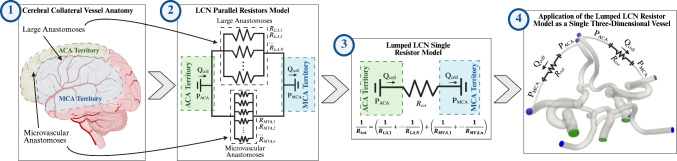
Schematic detailing the progressive simplification from (1) the complete anatomical LCN connecting the ACA and MCA territories, to (2) a parallel resistors model for all the collateral vessels, and ultimately to (3), a lumped single resistor model, which was applied in the three-dimensional model used in the present study (4) as a single vessel connecting the distal ACA and MCA in both hemispheres

Similarly, collateral vessels connecting the distal ACA to distal MCA with varying diameter (0.75 to 1.50 mm) were inserted on both the left and right side of the cerebral vasculature to mimic a range of poor to good patient collateralization for a patient with a thrombus located in the proximal M1-MCA, as shown in Fig. 1b. Although these additional anastomoses were not derived from CTA data, they were designed to be a consolidated and simplified model of the entire network of large anastomoses and pial arterial microvascular anastomoses connecting the distal ACA and MCA regions, which comprise most of the LCN responsible for the acute phase response following LVO to perfuse affected brain tissue and limit damage to the ischemic penumbra (Kim et al. 2021). This method for simulating the LCN was chosen because it facilitates an assessment of the cumulative macroscale hemodynamic effect across a spectrum of poorly to well-developed LCNs without having to mesh and solve for the flow within each individual collateral vessel. The latter would be computationally impractical given the size and quantity of the arteries and arterioles comprising the LCN. Angiography data of the LCN are also rarely acquired due to the spatial resolution limitations. Varying the diameter directly alters the hemodynamic resistance through the simulated LCN. Assuming Poiseuille flow through the additional collateral vessel, the collateral resistance increases as a function of $1/{D}^{4}$ based on the Hagen–Poiseuille equation. The lumped LCN model described above is summarized in Fig. 2.

This simple model allows us to sweep across a wide range of simulated LCN resistances that represent varying degrees of collateral development, since the total LCN resistance depends on the number of collateral vessels, vessel diameters, and the total length of each collateral vessel connection. Small diameter collateral vessels in the computational model represent individuals with poorly developed LCNs and greater collateral flow resistance, whereas larger collateral vessels represent individuals with a highly developed collateral network that more readily permits flow between the distal ACA and MCA brain regions. A summary of the anatomical collateral models created for the simulations and their respective total collateral resistances is included in Table 1. Each anatomy type is referred to by its respective simulated collateral score (i.e., CS0, CS1, etc.) listed in this table throughout this paper (Liu et al. 2018). The decision to focus on the ACA to MCA collaterals, omitting the PCA to MCA collaterals, was based on the findings of previous studies that suggest the ACA to MCA collaterals provide a larger contribution to compensatory MCA-dependent brain regions compared to the PCA to MCA collaterals (Coyle 1987; Brozici et al. 2003; Litman et al. 2025).

**Table 1:** Summary of the anatomical models used in this study with varying ACA to MCA collateral vessel diameters to represent poor to favorable collateral development levels that produce different levels of hemodynamic resistance through the vessel

Collateral Type	Collateral Score (CS)	Collateral Vessel Length (mm)	Collateral Vessel Diameter (mm)	Ipsilateral Collateral Resistance ($\times {10}^{9}$ $\mathbf{P}\mathbf{a}\bullet \mathbf{s}/{\mathbf{m}}^{3}$)
Absent	CS0	-	0	$\infty$
Small	CS1	35.5	0.75	18.3
Medium	CS2	35.5	1.00	5.8
Large	CS3	34.8	1.25	2.3
Extra-Large	CS4	34.8	1.50	1.1

Hemodynamic resistances were calculated using the Hagen–Poiseuille equation with input parameters defined based on the collateral vessel length and diameter, and the simulated blood dynamic viscosity (µ = 4 × 10^–3^ kg/(m•s))

### Computational fluid dynamics model governing equations

The continuity equation (Eq. 1) and a Darcy–Brinkman modified Navier–Stokes equation (Eq. 2) derived from mixture theory were used to model blood flow in the cerebral vasculature and to account for the added hemodynamic resistance and porosity within the simulated occlusive clot region (Bowen 1976). The model assumes incompressible and Newtonian behavior of the blood, which is a valid assumption for flow through these large vessels under typically high shear flow conditions. Fluid saturation is also assumed in the porous clot region (i.e., the fluid and solid volume fractions sum to one).

1 $$\nabla \cdot \left( {\Phi {\boldsymbol{u}}_{{\boldsymbol{f}}} } \right) = 0$$

2 $$\Phi \left(\frac{\partial {{\boldsymbol{u}}}_{{\boldsymbol{f}}}}{\partial t}+\left({{\boldsymbol{u}}}_{{\boldsymbol{f}}}\cdot \nabla \right){{\boldsymbol{u}}}_{{\boldsymbol{f}}}\right)=-\frac{\nabla p}{{\rho }_{f}}+{\nu }_{f}{\nabla }^{2}\left(\Phi {{\boldsymbol{u}}}_{{\boldsymbol{f}}}\right)-{\Phi }^{2}\frac{{\nu }_{f}}{{k}_{clot}}{{\boldsymbol{u}}}_{{\boldsymbol{f}}}$$

**Fig. 3:**
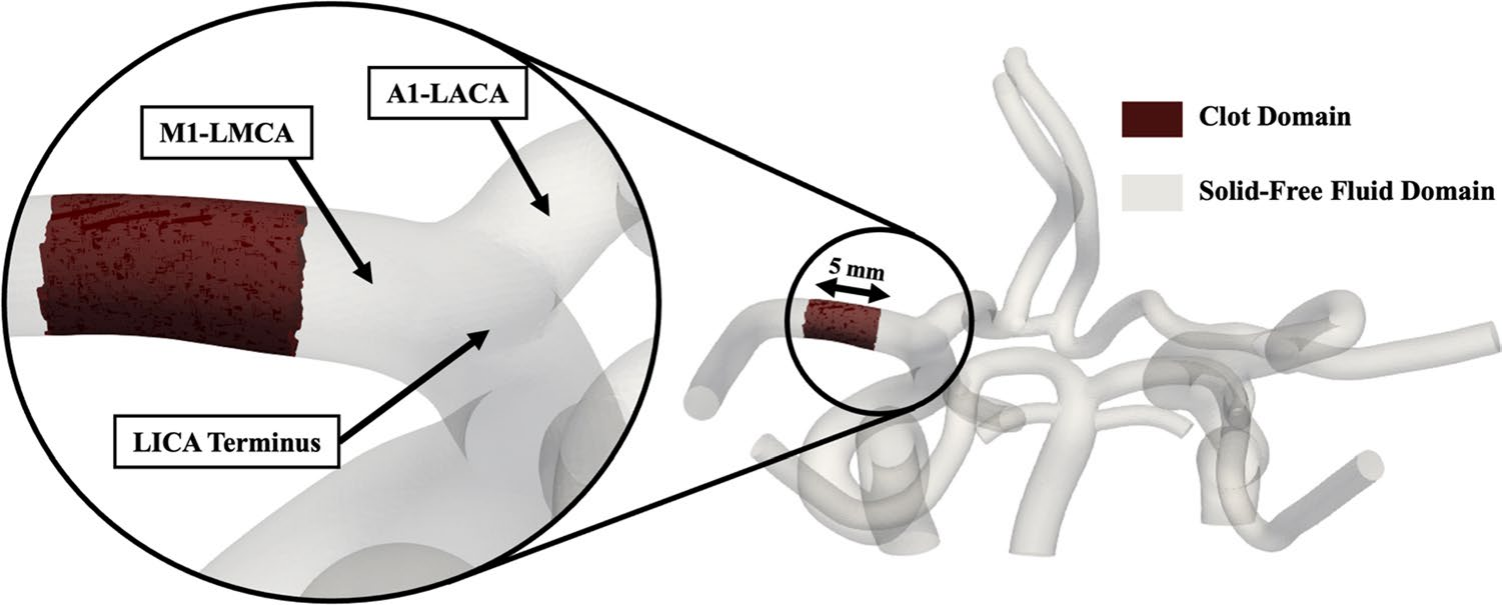
Visualization of the computational mesh with no collateral vessels highlighting the occlusive clot location and length in the proximal M1 segment of the LMCA to simulate LVO

In these equations $\Phi$ is the porosity (fluid volume fraction), ${{\boldsymbol{u}}}_{{\boldsymbol{f}}}$ is the fluid velocity vector, $p$ is the pressure, ${\rho }_{f}$ is the fluid density, ${\nu }_{f}$ is the fluid kinematic viscosity, and ${k}_{clot}$ is the permeability of the blood clot. The fluid density and kinematic viscosity were defined as 1060 kg/m^3^ and 3.77E × 10^–6^ m^2^/s (3.77 cSt), respectively, to match average blood properties. Within the computational domain, $\Phi <1$ when inside the clot domain and $\Phi =1$ when outside the clot domain. The blood clot was modeled as a porous fibrous media with isotropic permeability and a constant fiber diameter using the Davies’ equation (Eq. 3), developed from empirical data of air flow through a fibrous media and validated in fibrin clots (Davies 1953; Wufsus et al. 2013).

3 $${k}_{clot}=\frac{{R}_{f}^{2}}{16{\Phi }_{f}^{1.5}\left(1+56{\Phi }_{f}^{3}\right)}$$

where ${R}_{f}$ is the fibrin fiber radius, which was set to 250 nm to be consistent with the larger fibrin fiber diameters measured in clots formed in vitro with physiologically relevant thrombin and fibrinogen concentrations (Belcher et al. 2023), thus, meant to be representative microstructural properties of clots formed under in vivo environmental conditions. ${\Phi }_{f}$ is the fibrin fiber volume fraction, which was set to ${\Phi }_{f,0}=$ 0.18 to match the minimum fibrin volume fraction where the relationship between permeability and fibrin volume fraction plateaus and is well-described by the Davies’ equation (Wufsus et al. 2013; Hunter 2013). Based on these parameters, the simulated clot permeability was 0.0386 µm^2^. Although the actual permeability of blood clots in vivo is still largely unknown, this value falls within the permeability range of experimentally measured fibrin-dominant clots that were of the same size scale as the simulated clot in this study (Wufsus et al. 2013; Du et al. 2020). The clot was defined as a short 5-mm-long section of the proximal left M1-MCA, as shown in Fig. 3. Numerically this was achieved by setting ${\Phi }_{f}$ of the computational cells within the clot domain to be ${\Phi }_{f,0}$. Therefore, producing a hemodynamic resistance within those cells by making the Darcy term in the modified Navier–Stokes equation nonzero.

### Boundary conditions

**Fig. 4:**
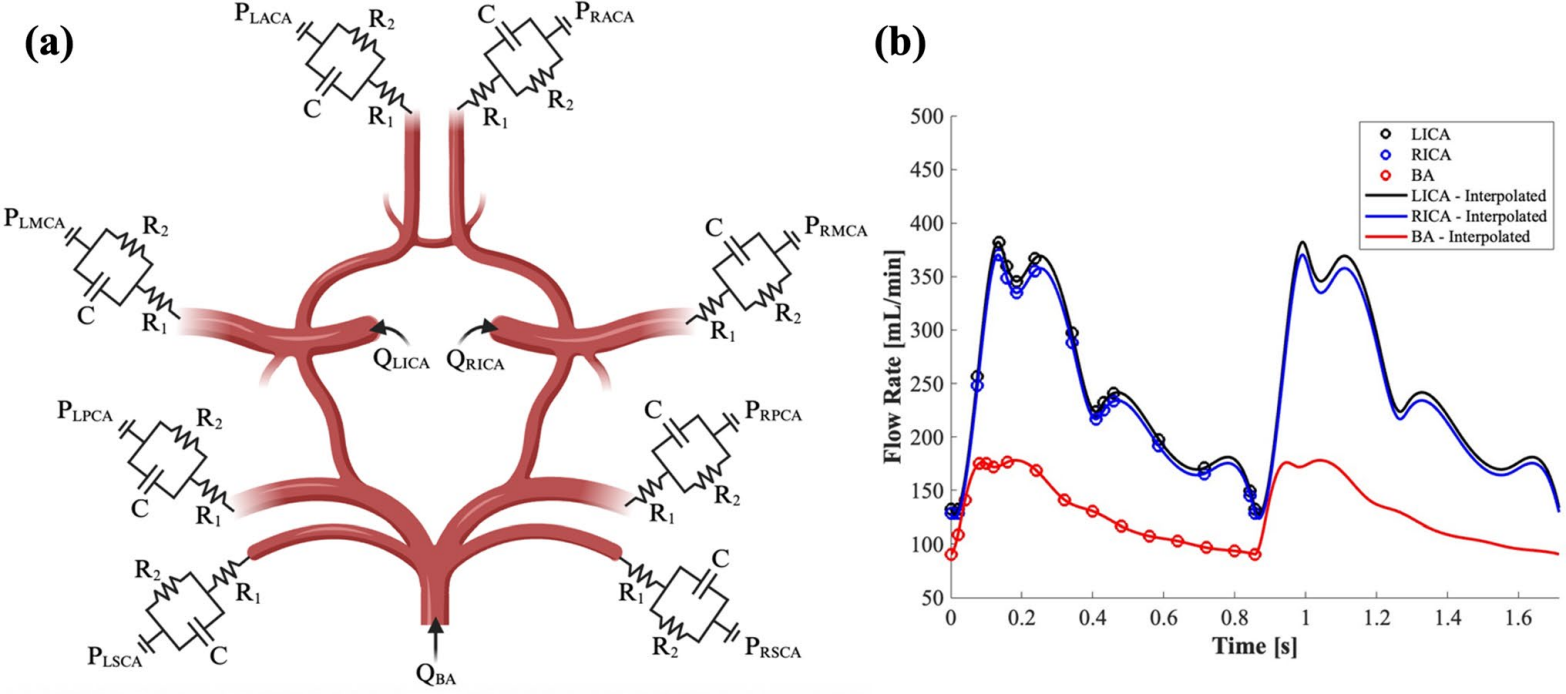
(**a**) Illustrative summary of the boundary conditions used in the CoW model, including the Windkessel boundaries to calculate boundary pressures and time-dependent inlet flow rates, and (**b**) line plots of the inflow waveform boundary conditions applied to the LICA, RICA, and BA (circles represent the original data derived from clinical PCMRI of elderly individuals (Hoi et al. 2010; Kim et al. 2017), and the lines are the interpolated waveforms used as the computational boundary conditions)

The boundary conditions used to simulate cerebrovascular hemodynamics in the model are summarized in Fig. 4. All three model inlets (LICA, RICA, and BA) were prescribed a time-dependent inflow velocity boundary condition meant to mimic physiological flow. ICA flow profiles were extracted and interpolated from phase contrast magnetic resonance imaging (PCMRI) data collected from healthy elderly demographic patients that do not have any vessel occlusions (Hoi et al. 2010). The BA inflow profile was extracted in a similar manner from (Kim et al. 2017). These inflow velocity profiles were then linearly scaled to match the mean inflow rate of the respective vessels for elderly adults that often experience AIS, and the total length of the cardiac cycle was set to 857 ms to achieve a simulated heart rate of 70 beats/min (Amin-Hanjani et al. 2015; Zarrinkoob et al. 2015). The discrete MRI data points were interpolated using a cubic spline method to improve the temporal scale by reducing the time step between data points toward the computational time step while maintaining the appropriate waveform shape. The interpolated waveform was repeated for each cardiac cycle over the course of the entire simulation time to replicate repeated heart beats. All the inlet boundary condition steps described were performed using an in-house MATLAB (MathWorks, Inc.; Natick, MA, USA) code. The resulting inlet flow rate boundary conditions compared to the MRI data from which they are derived are shown in Fig. 4b.

A three-element Windkessel model was used to govern the outflow and pressure at each vessel outlet to best match physiological pressure and flow conditions. The Windkessel model was parameterized based on existing estimations of peripheral resistance, distal resistance, and compliance that have been used previously in similar CFD models (Xiao et al. 2013). These parameters were iteratively modified to achieve: (1) mean arterial flow through the outlet vessels (MCA, ACA, PCA, and SCA) that closely matched clinical measurements of flow through the respective vessels in elderly individuals (Amin-Hanjani et al. 2015), and (2) systolic and diastolic pressures in the proximal inlet arteries (ICA and BA) that replicated typical systemic pressures of elderly individuals (Britton et al. 1986). Preliminary simulations were performed to calculate the relative flow distributions and pressures at each of the boundaries. The resistance and compliance parameters were adjusted linearly based on the relative difference between the computational and clinical values for flow rates and pressures, respectively. The simulations were then run again with the updated parameters to calculate the new relative differences. This process was repeated until all boundaries were within 10% of the target physiological values for mean flow rate, systolic pressure, and diastolic pressure. The final Windkessel parameters used in the model are summarized in Table 2. Pressure and flow waveforms along the boundaries were demonstrated to converge within the first two cardiac cycles using this Windkessel model.

**Table 2:** Final Windkessel model constitutive parameters for the outlet vessel boundaries. (${R}_{1}$—proximal resistance; ${R}_{2}$—distal resistance; $C$—compliance)

Parameter	Value
${R}_{1,LACA}$	$5.748\times {10}^{8} Pa\bullet s/{m}^{3}$
${R}_{2,LACA}$	$7.716\times {10}^{9} Pa\bullet s/{m}^{3}$
${C}_{LACA}$	$2.110\times {10}^{-11} {m}^{3}/Pa$
${R}_{1,LMCA}$	$3.096\times {10}^{8} Pa\bullet s/{m}^{3}$
${R}_{2,LMCA}$	$5.208\times {10}^{9} Pa\bullet s/{m}^{3}$
${C}_{LMCA}$	$6.329\times {10}^{-11} {m}^{3}/Pa$
${R}_{1,LPCA}$	$8.664\times {10}^{8} Pa\bullet s/{m}^{3}$
${R}_{2,LPCA}$	$7.776\times {10}^{9} Pa\bullet s/{m}^{3}$
${C}_{LPCA}$	$3.164\times {10}^{-11} {m}^{3}/Pa$
${R}_{1,RACA}$	$5.748\times {10}^{8} Pa\bullet s/{m}^{3}$
${R}_{2,RACA}$	$7.716\times {10}^{9} Pa\bullet s/{m}^{3}$
${C}_{RACA}$	$2.110\times {10}^{-11} {m}^{3}/Pa$
${R}_{1,RMCA}$	$3.024\times {10}^{8} Pa\bullet s/{m}^{3}$
${R}_{2,RMCA}$	$5.040\times {10}^{9} Pa\bullet s/{m}^{3}$
${C}_{RMCA}$	$6.329\times {10}^{-11} {m}^{3}/Pa$
${R}_{1,RPCA}$	$8.664\times {10}^{8} Pa\bullet s/{m}^{3}$
${R}_{2,RPCA}$	$7.776\times {10}^{9} Pa\bullet s/{m}^{3}$
${C}_{RPCA}$	$3.164\times {10}^{-11} {m}^{3}/Pa$

### Numerical implementation

The governing equations were programmed into a custom solver using the open-source CFD package OpenFOAM v7.0 (OpenFOAM Foundation Ltd.; London, UK). Initial code verification simulations were run for a simple canonical case to confirm the numerical implementation of the CFD equations converged onto the analytical solutions. Details of the code verification study are included in Appendix A. The computational meshes were created in CF-MESH + 4.5.1 (Creative Fields; London, UK). An initial grid independence study was performed on the CS4 anatomical model and an occlusive M1-LMCA clot. To perform the grid independence study, three separate meshes were generated with a grid refinement ratio of 1.5. The Roache grid convergence index (GCI) was then calculated with total pressure force applied to the clot and the ipsilateral collateral flow rate as the output parameters of interest and a safety factor of 1.25. The calculated GCI error band for the force and flow results was 0.21 and 0.90%, respectively. The low discretization error band estimate of the GCI indicated computational solution grid independence and accuracy. The solutions were also verified to be within the asymptotic range of grid convergence by calculating the ratio of the GCI for the fine and coarse meshes and confirming it met the prescribed criteria, as described by Roache (Roache 1994). Details of the grid independence study are included in Appendix B. The final meshes were composed of between 593,542 and 634,941 cells, dependent on the presence and size of the collateral vessels. Each mesh had two additional boundary cell layers added to refine the near-wall cells and to control the first boundary cell size. The first cell maximum size was set to 0.14 mm, to maintain a y + value less than 1 based on the inlet diameters and maximum free-stream velocities.

The computational model was coded as a transient pressure-based solver, which used a merged pressure-implicit with splitting of operators (PISO) and semi-implicit method for pressure linked equations (SIMPLE) algorithms for pressure–momentum coupling, similar to the built-in pimpleFoam solver in OpenFOAM. Three SIMPLE outer corrector loops and four PISO inner corrector loops were completed for each time step with a pressure and velocity under-relaxation factor of 0.3 and 0.7, respectively, for smooth convergence. The computational time step was modified throughout the simulation, so the maximum Courant number was 5, resulting in an average Courant number less than 1 across the whole computational domain during the entire cardiac cycle. A second-order implicit backward time differencing was used for the temporal term, and a second-order linear spatial differencing was used for the convective and diffusive terms to numerically solve the modified Navier–Stokes equation. The final Darcy term was added as an implicit source. Pressure and velocity residuals were converged to 10^–6^ and 10^–9^, respectively, for each time step. Simulations were run for 6 s of computational time for each anatomy both with and without an occlusive clot in the M1-LMCA, resulting in 7 complete cardiac cycles for pressure and flow analysis. Each simulation took an average of 90 min to complete while computing in parallel on 256 processor cores of the Anvil high-power computing cluster at Purdue University (Hacker et al. 2014; Song et al. 2022; Boerner et al. 2023).

### Data post-processing

All the hemodynamic data were extracted from the computational simulations using the post-processing utility built into OpenFOAM. The pressure waveforms in each major CoW vessel were generated by calculating the average pressure across all the faces along the corresponding vessel outlet boundary at each time point. The flow rates were calculated by summing the volumetric flux through all the boundary faces for each corresponding vessel. Mass continuity was verified within the system by confirming the sum of volumetric flow at the inlets matched that of the outlets. The collateral flow was calculated by creating separate internal baffles at the center of both the ipsilateral and contralateral collateral vessels and summing the volumetric flow through all the computational cell faces comprising each baffle. The pressures proximal and distal to the clot were recorded using the probes function object in OpenFOAM to sample the pressure at points within the fluid domain that are approximately 1 mm away from the proximal and distal clot faces. Figure generation and post-processing of the extracted pressure and flow data, including calculations of the mean arterial pressures (MAP) and flow rates, were performed using an in-house MATLAB code. The MAP and mean flow rate (${Q}_{Mean}$) were calculated using Eqs. 4 and 5.

4 $$\mathrm{MAP}= \frac{2{P}_{Dia}+{P}_{Sys}}{3}$$

5 $${Q}_{Mean}=\frac{1}{T}{\int }_{{t}_{0}}^{{t}_{0}+T}Q(t)dt$$

where ${P}_{Dia}$ and ${P}_{Sys}$ are the diastolic and systolic pressures in the vessel of interest, respectively, $T$ is the period of the cardiac cycle, and $Q(t)$ is the extracted blood flow rate in the vessel of interest at time $t$. MAP and flow rate calculations excluded the first two cardiac cycles that may be affected by the time-dependent Windkessel initialization at the outlets. Velocity streamline figures were generated in ParaView 5.10.1 (Kitware, Inc.; Clifton Park, NY, USA).

## Numerical results

### Baseline CoW Hemodynamics pre-occlusion

**Fig. 5:**
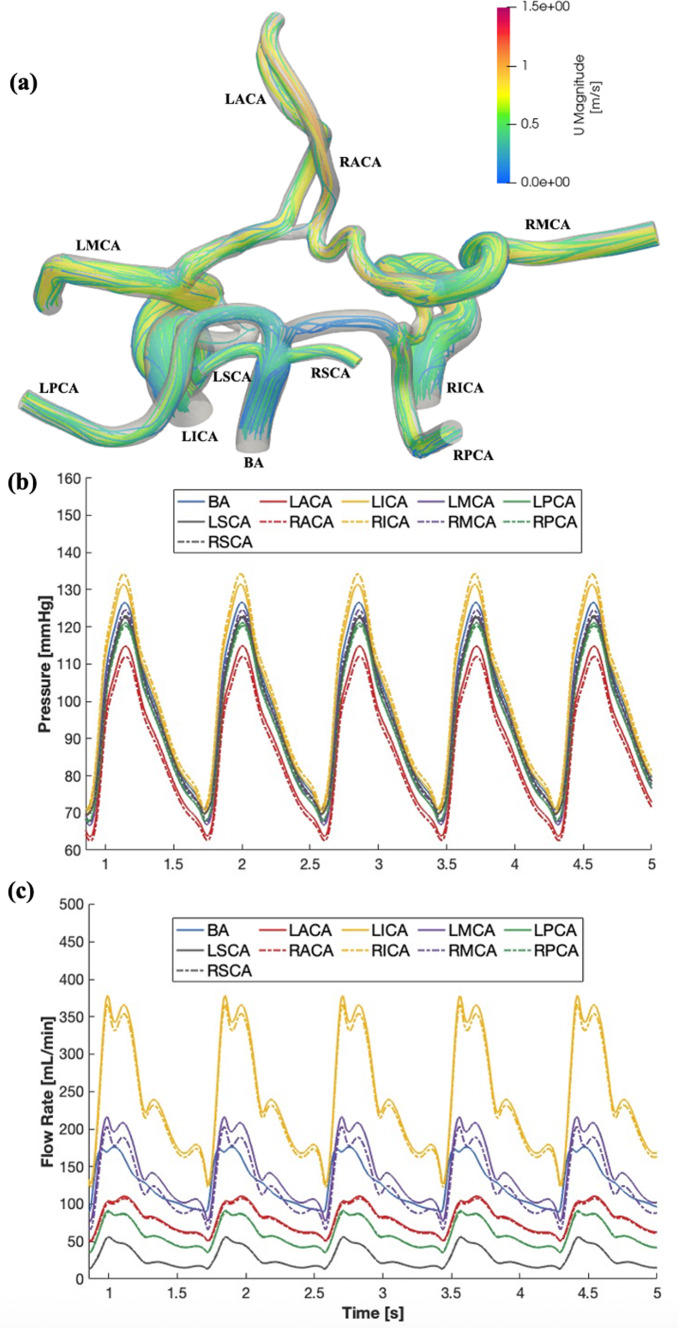
(**a**) Representative velocity streamline plots shown through the CS0 (no collaterals) CoW anatomy in a healthy control case with no occlusive blood clot, and line plots of the corresponding (**b**) pressure and (**c**) flow waveforms at all the major inlets and outlets of the anatomy

The average flow rate through each outlet vessel in a healthy control state with no occlusive clot present matched well with the clinical PCMRI data taken from healthy elderly patients. The maximum percent error was less than 9%, and all values were within one standard deviation from the clinical mean value (Amin-Hanjani et al. 2015; Zarrinkoob et al. 2015). Comparisons of the mean flow rates calculated in all the major CoW vessels to in vivo PCMRI data are included in Table 3. Information regarding flow through the SCAs was not included in the clinical dataset and could therefore not be explicitly validated. However, the agreement between clinical and computational flow rates in the three other vessels comprising the posterior circulation (BA, RPCA, and LPCA) suggests that the total SCA flow in the computational model matches well with the flow in their respective vessels in vivo. There were negligible differences in the CBF distribution among the five anatomical models tested, as shown in Table 4. The largest differences were seen in the ACA and MCA, whose mean flow rate varied only by 1.5 mL/min when comparing the CS0 and CS4 anatomies. A representative visualization of flow streamlines through the anatomy, and pressure and flow waveforms is shown in Fig. 5. In general, these streamlines show higher blood flow velocities in the anterior vessel compared to the posterior vessels, with velocities increasing toward the outlets as the vessels taper to smaller diameters. They also illustrate the complex flow patterns that arise in these vessels due to the vessel tortuosity and branching that can vary greatly depending on individual anatomy. The flow and pressure waveforms (Fig. 5b, c) demonstrate that the time-dependent inlet flow rate boundary conditions, in conjunction with the outlet Windkessel pressure boundary conditions, generate physiologically relevant pulsatile pressure and flow within the major CoW vessels that are representative of those in vivo.

**Table 3:** CFD results in the CS0 anatomy with no occlusive clot compared to clinical cerebrovascular flow data measured in healthy elderly patients using PCMRI (Amin-Hanjani et al. 2015)

Inlet Boundary	In Vivo PCMRI Flow Data (mL/min)	Computational Flow Results–CS0 (mL/min)	Percent Error (%)
LICA	243 $\pm$ 53	243.07	0.0%
RICA	235 $\pm$ 53	235.25	0.1%
BA	124 $\pm$ 31	127.05	2.5%

Outlet Boundary	In Vivo PCMRI Flow Data (mL/min)	Computational Flow Results–CS0 (mL/min)	Percent Error (%)
LACA	79 $\pm$ 26	80.65	2.1%
LMCA	146 $\pm$ 22	142.76	2.2%
LPCA	61 $\pm$ 12	60.02	1.6%
LSCA	-	28.12	-
RACA	86 $\pm$ 32	78.83	8.3%
RMCA	133 $\pm$ 26	126.80	4.7%
RPCA	58 $\pm$ 12	59.63	2.8%
RSCA	-	28.20	-

**Table 4:** Summary of mean arterial flow rate (MAQ) and pressure (MAP) at the major vessel boundaries for each of the anatomical models simulated without an occlusive clot in the system

Vessel Boundary	CS0		CS1		CS2		CS3		CS4	
	MAQ [mL/min]	MAP [mmHg]	MAQ [mL/min]	MAP [mmHg]	MAQ [mL/min]	MAP [mmHg]	MAQ [mL/min]	MAP [mmHg]	MAQ [mL/min]	MAP [mmHg]
LICA	243.1	90.3	243.1	90.4	243.1	90.4	243.1	90.3	243.0	90.3
RICA	235.3	92.1	235.2	92.1	235.2	91.9	235.2	91.7	235.2	91.5
BA	127.1	89.4	127.1	89.4	127.1	89.3	127.1	89.2	127.1	89.1
LACA	80.7	80.8	80.7	80.9	80.9	81.1	81.2	81.4	81.5	81.7
LMCA	142.8	85.3	142.7	85.3	142.7	85.3	142.6	85.3	142.5	85.2
LPCA	60.0	85.5	60.0	85.5	60.0	85.5	59.9	85.4	59.9	85.3
LSCA	28.1	87.3	28.1	87.2	28.1	87.2	28.1	87.1	28.0	87.0
RACA	78.8	79.1	78.9	79.2	79.3	79.6	79.8	80.0	80.4	80.6
RMCA	126.8	86.7	126.7	86.6	126.3	86.4	125.8	86.0	125.2	85.6
RPCA	59.6	85.4	59.6	85.4	59.6	85.3	59.5	85.2	59.4	85.1
RSCA	28.2	87.4	28.2	87.4	28.2	87.4	28.1	87.3	28.1	87.2

The simulated total cerebral blood flow (CBF) was 605.4 mL/min, of which 79% came from the anterior vessel inlets (LICA and RICA) and 21% from the posterior vessel inlet (BA). At the outlets, this anterior–posterior CBF balance remained similar to the inlets, with 71% of flow exiting the anterior outlets (ACAs and MCAs) and 29% to the posterior outlets (PCAs and SCAs). Thus, in the healthy control state with no occlusion, the PComAs do not play a role in redistributing flow between the posterior and anterior circulations. This is also apparent when evaluating the streamlines in Fig. 5a, where flow appears to be less prevalent in these arteries compared to the other major vessels. There was also a slightly greater amount of blood flow distributed to the left hemisphere cerebral vessels (46.8%) compared to the right (43.8%). This slight distribution disparity between hemispheres is also observed in clinical data (Amin-Hanjani et al. 2015). The left and right MCA received the largest fractions of the CBF distribution. The LMCA outflow comprised nearly 24% of the total CBF, and the RMCA nearly 21%. The large percentage of blood flow into these vessels likely contributes to the MCA being the most common AIS occurrence location (Liu et al. 2018). The high flow distribution to the MCAs also suggests that flow reduction or cessation due to vessel occlusion will have the greatest impact on cerebral hemodynamics.

The systemic systolic (sSBP) and diastolic (sDBP) blood pressures, estimated as the average maximum and minimum pressures in the three proximal inlet vessels (LICA, RICA, and BA), were 130.5 and 70.4 mmHg, respectively, resulting in a simulated systemic MAP of 90.4 mmHg. These values are slightly lower than the typical sSBP, sDBP, and systemic MAP values measured in elderly individuals, which are approximately 135, 83, and 100 mmHg, respectively (Gupta et al. 2013). However, the model is still expected to be a good representation of cerebral hemodynamics because pressure in the large proximal cerebral vessels is slightly lower compared to that of the brachial artery where systemic pressure is measured typically. This difference arises due to the greater pressure losses from the smaller diameters and increased tortuosity of the superior vessels (Shoemaker et al. 2025). This is also reflected here because the smallest and most distal vessel outlets, such as the ACA and PCA, had the lowest pressures compared to larger and more proximal vessels, such as the MCA and SCA.

The baseline healthy control simulations demonstrated that our model was able to recapitulate bulk cerebrovascular hemodynamics, including physiological pressure waveforms and flow distributions within the CoW. These control simulations also showed that LCN development level did not alter the CoW hemodynamics appreciably when none of the vessels were occluded.

### Altered CoW hemodynamics following MCA occlusion

**Fig. 6:**
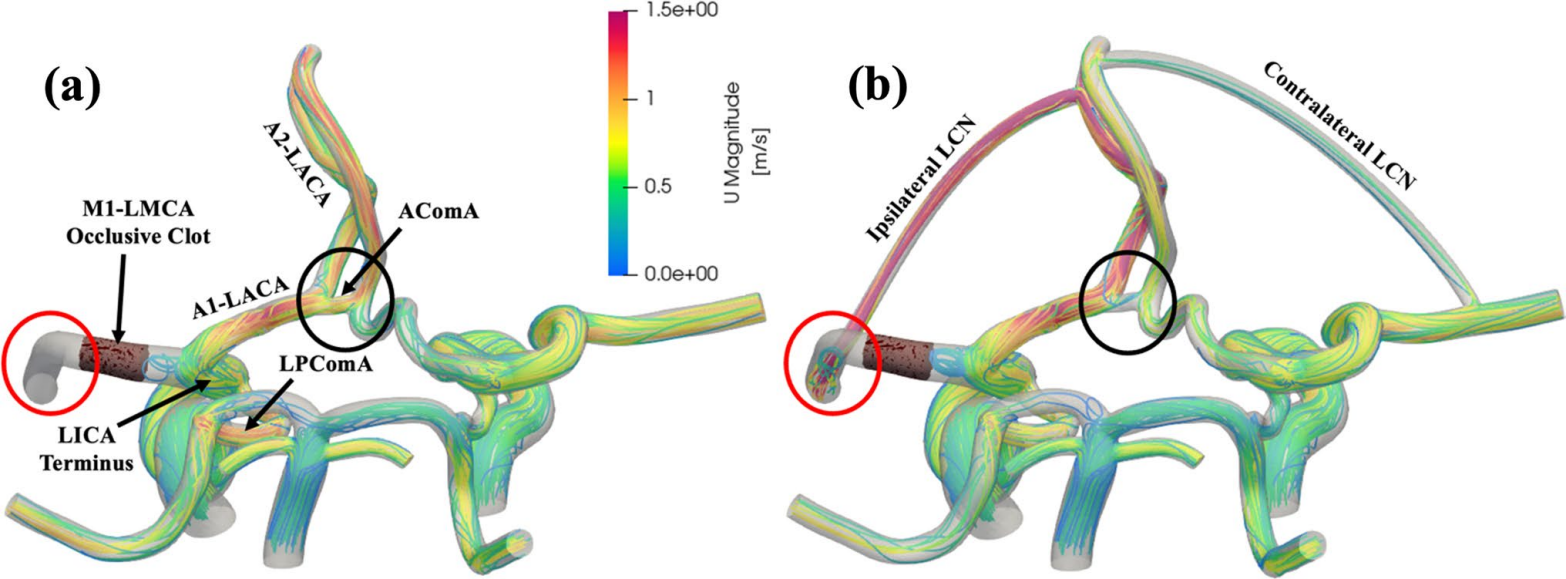
Comparison between representative streamlines for anatomical models (**a**) CS0 and (**b**) CS4 with an occlusive M1-LMCA clot (red circles highlight the contrast in MCA flow distal to the clot between the CS0 case and the CS4 case, and black circles highlight the decreased flow from the LACA to the RACA through the AComA in the CS4 case)

Cases with the simulated M1-LMCA occlusion showed a drastic decrease in LMCA flow, as expected due to the large hemodynamic resistance introduced by the clot. In the CS0 anatomy, there was near zero (< 0.01 mL/min) flow through the LMCA because of this elevated resistance at the occlusion site and the lack of collaterals that are meant to compensate for this lack of distal MCA flow, highlighted by the red circle in Fig. 6a. The blood flow was diverted away from the LMCA to the proximal A1-LACA and the LPComA since these are the other vessels near the LICA terminus. The velocity magnitude and the local streamline density increased in each of these vessels. The anatomical models with collateral vessels (CS1-CS4) showed an increased high velocity flow through the collaterals following occlusion, which led to observed flow distal to the clot in the LMCA, highlighted by the red circle in Fig. 6b. In all cases, the flow velocity through the A1-LACA again increased. Unlike the CS0 case, these cases did not have as significant of an increase in AComA flow, highlighted by the black circles in Fig. 6, because more flow was able to pass to the distal A2-LACA due to the presence of the collateral vessel anastomosis at this distant vessel.

**Fig. 7:**
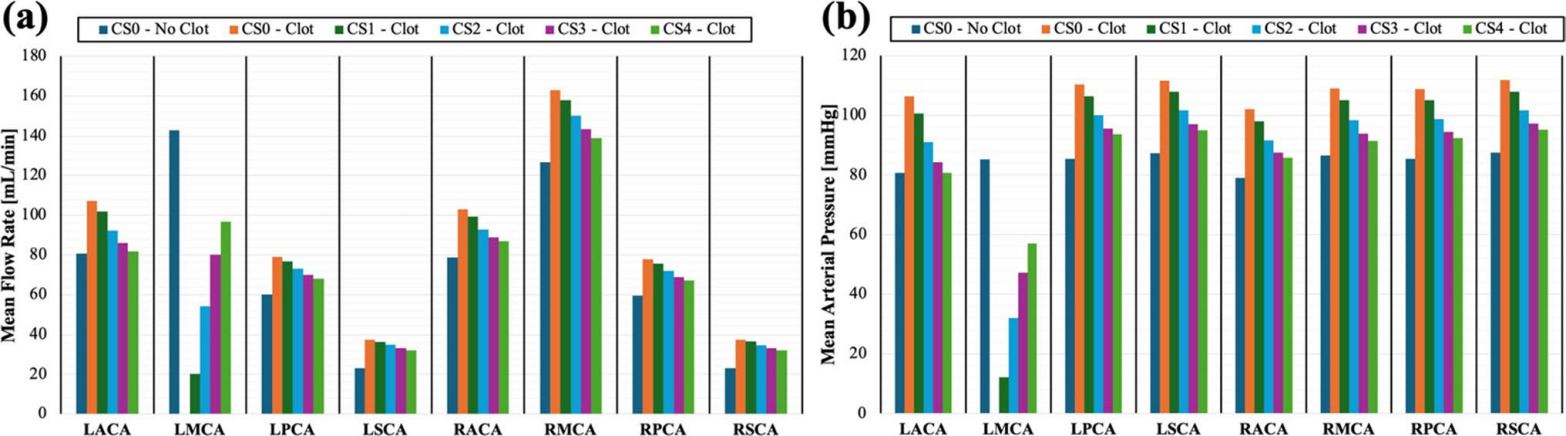
Bar graphs showing the distribution of (**a**) mean flow rate and (**b**) MAP for each of the outlet vessels in all five anatomical models with the M1-LMCA occlusive clot (CS0-CS4–Clot) compared to a representative case without the clot present (CS0–No Clot)

Figure 7a shows the mean flow distribution across the eight outlet vessels for all five anatomical models with an occluded M1-LMCA compared to a representative case without the occlusion (CS0–No Clot). Flow remained relatively well-balanced between the LACA and RACA outlets despite the increased blood flow into the proximal A1-LACA. This indicated that for CS0 with an MCA occlusion the increased A1-LACA flow was balanced by increased flow through the AComA into the RACA, and for the cases with collaterals (CS1-CS4) the flow was balanced by diversion through both the AComA and the ipsilateral collateral vessel. These phenomena demonstrate the primary and secondary collateral capacity of the cerebral vasculature that attempts to maintain normal physiological flow in instances of disturbances due to LVO. As the collateral size increased from CS1 to CS4, the LMCA flow increased because the flow compensation capacity was greater in the larger collateral vessel with lower hemodynamic resistance.

Figure 7b shows the MAP in the outlet vessels for each of the anatomical models compared to that of the healthy control case. Following LMCA occlusion, there was a pressure increase in all the vessels except in the occluded vessel, where the clot led to a steep pressure drop. This phenomenon was most pronounced in the CS0 anatomical case, in which MCA occlusion caused a roughly 30% MAP increase in all the vessels except the LMCA where the pressure dropped to near zero. As the collateral vessel size increased from CS1-CS4, the MAP in all the vessels shifted closer to their respective healthy control MAP, including in the occluded MCA because of the connection between the distal LMCA and LACA. The occluded cases also had an elevated pulse pressure compared to the non-occluded cases. The non-occluded cases had a pulse pressure of 60.2 mmHg, and the pulse pressure in the occluded cases ranged from 83.1 to 88.8 mmHg depending on the collateral development level. The CS2 case had the highest pulse pressure, and the CS1 case had the lowest pulse pressure. The altered pulse pressure was a result of the systolic pressure increasing more than the diastolic pressure following occlusion.

### Collateral recruitment effect following occlusion

The ipsilateral collateral flow rate greatly increased following M1-LMCA occlusion. The largest increase in flow rate relative to the flow rate in the healthy case pre-occlusion was observed in the CS1 anatomy, where the ipsilateral collateral flow increased over 20-fold from 1.01 to 20.33 mL/min. Despite the high resistance through the small vessel, the steep pressure gradient driving flow from the ACA to the distal MCA was sufficient to drive this large flow rate increase. Each of the other anatomies also demonstrated significant flow increases compared to the non-occluded case. CS2 had over a 14-fold increase, CS3 had over ninefold increase, and CS4 had over a sevenfold increase, as shown in Table 5. In comparison, the contralateral collateral vessel illustrated very little change following occlusion. Across all anatomies, the contralateral collateral vessel did not change by more than 2 mL/min, which corresponded to a negligible 1.09-fold increase from the healthy flow. This emphasizes that the collateral recruitment effect is largely focused in the ipsilateral hemisphere.

**Table 5:** Collateral circulation recruitment following MCA occlusion and the influence of more highly developed collateral networks increase flow through the collateral circulation to the distal MCA

Anatomy	Ipsilateral Collateral Flow (mL/min)	Ipsilateral Flow Ratio (Occluded/Non-Occluded)	Compensation of MCA Flow Without Occlusion	Contralateral Collateral Flow (mL/min)	Contralateral Flow Ratio (Occluded/Non-Occluded)
CS1	20.33	20.13	14.2%	1.49	0.91
CS2	53.91	14.04	37.8%	5.73	0.94
CS3	79.89	9.64	56.1%	13.09	1.03
CS4	98.21	7.32	69.0%	21.58	1.09

As mentioned previously, increased diameter of the collateral vessel in the model reduced hemodynamic resistance and increased the compensatory capacity of the simulated LCN. This effect was observed here, as the mean ipsilateral collateral flow rate increases with collateral vessel diameter. The ipsilateral collateral flow rate during MCA occlusion in the CS1 anatomy was 20.33 mL/min, which compensated for 14.2% of the original LMCA flow rate without the occlusion. At the other extreme in the CS4 anatomy, the ipsilateral collateral flow rate increased to 98.21 mL/min, which was 69.0% of the original MCA flow rate. The CS1 and CS2 anatomies were able to compensate for 37.8 and 56.1% of the healthy LMCA flow, respectively. The rate at which the collateral flow compensatory capacity increased with the vessel diameter was smaller at the larger vessel sizes, indicating a plateauing effect at the larger vessels. This is likely due to a decline in the pressure difference between the distal ACA and MCA, which leads to smaller pressure gradients driving flow in the larger vessel cases.

### Effect of collateral development level on pressure drops across the clot and distal compaction forces

The introduction of collaterals into the system decreased the pressure difference between the proximal and distal end of the clot. The pressure difference reduction was a combined result of decreasing systemic pressure and increasing distal MCA pressure, both of which were dependent on the diameter of the collateral vessel. Details of the relationship between collateral development level and the pressure difference are summarized in Table 6. The mean proximal pressure did not change as significantly with collateral development level as the mean distal pressure. Mean proximal pressure decreased by only 18.7 mmHg between the two collateral size extremes from 117.8 mmHg in the CS0 anatomy to 99.2 mmHg in the CS4 anatomy, whereas the mean distal pressure rose by 58.8 mmHg from the CS0 to CS4 anatomy, which indicates that collateral modulation of the distal pressure in the occluded vessel plays a greater role in dictating the pressure gradient across the clot. All the computational proximal mean pressures were within the upper range of the 95% confidence interval of clinical data taken proximal to an occlusive clot during AIS and the distal mean pressures spanned nearly the entire confidence interval range of the clinical data. The predicted mean pressure differences between the proximal and distal end were also representative of the pressure differences observed in vivo. The computational pressure difference range across all the simulated anatomies matched closely with that of the clinical data confidence interval. In general, the CS2 anatomical cases matched best with the average clinical data. There was only an average difference of 5.7% when comparing the predicted proximal and distal pressure values to the measured clinical data. The only simulated case that fell outside the clinical confidence interval for both distal pressure and pressure difference across the clot during MCA occlusion was the CS0 anatomy. The complete lack of collaterals in this simulated case caused an extreme pressure drop in the distal vessel that was unlikely to be observed in real patients.

**Table 6:** Influence of collateral network on the proximal pressure, distal pressure, and pressure difference across the M1-LMCA thrombus compared to in vivo data measured in stroke patients (Sorimachi et al. 2011)

	Collateral Development Level	Proximal MAP (mmHg)	Distal MAP (mmHg)	Mean $\Delta$ P (mmHg)	F_mean_ (mN)	F_max_ (mN)
Absent	In Silico* –* CS0	117.8	0.0	117.8	116.3	172.7
Poor	In Silico* –* CS1	113.3	12.4	100.9	101.3	154.5
$\downarrow$	In Silico* –* CS2	106.5	33.0	73.2	77.0	126.1
	In Silico* –* CS3	101.5	48.8	52.3	58.7	99.4
Favorable	In Silico* –* CS4	99.2	58.8	39.6	47.8	82.5
	In Vivo – Mean (95% C.I.) [3]	95.8 (69.8–121.8)	32.7 (3.7–61.7)	63.1 (24.9–101.3)	58.0 (22.9–93.1)	-

The mean and maximum net distal compaction forces on the clot due to the pressure differences (${F}_{mean}$ and ${F}_{max}$) were calculated using Eqs. 6 and 7.

6 $${F}_{mean}={MAP}_{prox}{A}_{prox}-{MAP}_{dist}{A}_{dist}$$

7 $${F}_{max}={P}_{sys,prox}{A}_{prox}-{P}_{sys,dist}{A}_{dist}$$

where ${MAP}_{prox}$ and ${MAP}_{dist}$ are the MAPs proximal and distal to the clot, ${A}_{prox}$ and ${A}_{dist}$ are the proximal and distal cross-sectional clot face areas (7.40 and 6.38 mm^2^), and ${P}_{sys,prox}$ and ${P}_{sys,dist}$ are the peak systolic pressures proximal and distal to the clot. Because proximal and distal clot face areas were not available with the clinical data, in vivo forces were estimated based on the pressure difference across the clot multiplied by the mean of the proximal and distal clot face areas within the computational model.

In general, the lower pressure differences between the proximal and distal end of the clot due to increased collateral vessel diameter led to a linear decline in distal compaction forces experienced by the clot, as shown in Table 6. The mean distal compaction force on the clot for anatomical case CS0 and CS4 were 116.3 and 47.8 mN, respectively. The predicted mean distal compaction force in anatomical case CS3 was closest to that of the in vivo data, with only a 1.3% difference between the two values.

## Discussion

In the present study, we developed a model to replicate physiological flow within the major cerebral vasculature of elderly individuals and to capture hemodynamic changes within the CoW following MCA occlusion, simulating AIS. We also validated this model with clinical flow and pressure data in both healthy control and AIS cases by showing that the computational results for arterial pressures matched clinically measured data and CBF distributions were well below the 10% error tolerance compared to the average flow distributions observed clinically in the major vessels of the CoW. Leveraging this computational model platform, we studied how collateral development in an individual patient can impact specific factors that are influential to therapeutic outcomes for AIS patients treated with recanalization therapies, including IVT and EVT.

### Validity of the single collateral vessel model as a surrogate for modeling the entire LCN

The complex LCN was simulated using a simplified, single collateral vessel between the ACA and MCA (Fig. 2). LCN development was varied simply by modifying the collateral vessel diameter to change the hemodynamic resistance between the ACA and MCA within the system. In this manner, the simplified model can be representative of the entire complex anatomy relative to the arterial scale hemodynamics. Although it is impossible to determine the exact hemodynamic resistance of the entire LCN in vivo due to the network extent, vessel size, and individual variability, estimates can be made based on data from anatomical cadaver studies of the LCN along with assumptions regarding the vessel size and length distributions within the LCN. A previous study of the cerebral vessels in human cadavers by Eecken et al. (1953) found that there is an average of 11 larger secondary anastomoses connected to the MCA with diameters ranging from 200 to 610 µm. Estimating the average anastomosis length to be 2 cm and diameter to be 350 µm, the combined hemodynamic resistance from these vessels calculated using the Hagen–Poiseuille equation combined with a parallel resistors model is 1.97 × 10^10^ $\mathrm{Pa}\bullet \mathrm{s}/{\mathrm{m}}^{3}$, which is on the order of the smallest simulated collateral vessel case (CS1), meaning that the CS1 case is a representative example of a patient with very poorly developed LCN that is comprised only of these larger anastomoses and there is no contribution from the pial microvascular anastomoses or penetrating arteries. Taking a similar parallel resistors approach to relate our computational anatomical models with real-world anatomy, when accounting for the smaller microvascular anastomoses, the total resistance of the LCN will further decrease toward the other simulated cases with lower collateral hemodynamic resistance. Here, we assume a uniform small anastomotic vessel distribution with an average diameter and length of 60 µm and 5 mm, respectively (Duvernoy et al. 1981). To reduce the total hemodynamic resistance to that of the largest simulated collateral vessel case (CS4), the total number of anastomotic connections for each brain hemisphere would have to be roughly 50,000. This seems like a reasonable estimation for individuals with a highly developed LCN and is on the order of the number of small anastomotic connections that have been previously estimated in a computational model of the whole brain arterial network (Otani et al. 2023). Therefore, we believe this simplified method for simulating the entire LCN using one variable collateral vessel is valid from a bulk hemodynamics perspective.

### Comparison to current computational fluid dynamics models for cerebral flow

Our model provides a few distinct advantages over existing computational models. First, the inflow waveforms governing the time-dependent total CBF can be modified to match the flow waveforms observed in clinical data. This will be critical for assessing how patient-specific factors that can influence CBF, such as aging and heart disease, may impact AIS outcomes. The Windkessel model governing the outlet boundaries is easily tunable based on the resistance and compliance input parameters. Parameter tuning at the outlets permits the model to match pressure and flow distributions to patient-specific data. The model can also be applied to different CoW reconstructions to simulate flow and pressure changes in patient-specific anatomies. Finally, the model simulates vessel occlusion due to embolus lodging in a more realistic manner compared to previous computational studies. Previous studies have simulated AIS by assuming zero flow through the occluded vessel by applying infinite resistance (Padmos et al. 2021) or by including a vessel stenosis at the occlusion site to increase resistance (Kennedy McConnell and Payne 2017). While these methods may alter the surrounding hemodynamics in a manner similar to an occlusive clot, they are simplifications of the behavior of the permeable clot. The continuum-based Darcy–Brinkman method introduced here combined with the Davies equation approach to calculating clot permeability and mapping clot location to specific computational cells allows us to directly simulate a porous clot in the vasculature whose permeability is dependent on the physical microstructural properties of the clot. As such, we can leverage this model in the future to simulate different clot types, locations, and occlusion fractions to see how they influence cerebral hemodynamics during stroke. All these features will be important in the development of a reliable computational platform for evaluating hemodynamic responses to AIS to ultimately lead to better patient-specific treatment planning and outcome prediction approaches.

Previous computational models predicting flow changes within the CoW due to altered patient hemodynamics or altered cerebral vessel anatomy have largely ignored effects of secondary collateral flow (Alastruey et al. 2007; Šutalo et al. 2014; Darvish et al. 2021; Yang et al. 2024; Straccia et al. 2024). The few models that have looked at collateral flow have mostly used one-dimensional models to replicate the LCN and predict how LCN development influences distal brain perfusion following LVO (Kennedy McConnell and Payne 2017; Padmos et al. 2021; Otani et al. 2023; Epp et al. 2023). These studies have not incorporated realistic three-dimensional anatomical models of the CoW and have not assessed the changes to arterial scale hemodynamics and how they relate to the mechanisms of IVT and EVT therapy success. Our model has lower fidelity in the collateral system because it uses the single vessel lumped collateral model compared to the vast network of many small arterioles, but higher fidelity in the major CoW vessels because we included a realistic anatomical model. Therefore, we lose out on the nuances of the distal CBF and brain region perfusion distributions but can better predict macroscale blood flow effects that are more critical to therapy success.

### Hemodynamic changes across collateral development levels and AIS conditions

Our model demonstrated that under normal physiological flow without the presence of an occlusion, the LCN has little effect on blood flow through the CoW regardless of the LCN development since each collateral vessel size produced roughly the same pressure and flow through each of the major vessels. These findings are consistent with clinical observations of the LCN being relatively inconsequential to blood flow distributions between arterial territories under healthy conditions due to the antegrade bidirectional flow away from the CoW (Uniken Venema et al. 2022a). However, following acute vessel occlusion, the LCN development has a significant effect on the CoW hemodynamics. Our study showed there is a relationship between the collateral development and hemodynamic parameters such as collateral flow rate, systemic pressure, flow distributions in the major vessels, and pressure drop across the occlusive clot.

The results of our study found that the newly developed pressure gradient from the distal ACA to the distal MCA drove more unidirectional retrograde flow from the patent ACA brain territory to the occluded MCA brain territory. Figure 6 highlights the increased blood flow velocity in the ipsilateral collateral vessel, which caused higher shear stresses along the vessel wall. The elevated wall shear stresses initiate a positive feedback loop to increase collateral compensatory capacity through endothelial cell-mediated vasodilation of the LCN vessels. Collateral recruitment through this signaling pathway can be thought of as a progression from one of the poor collateral development cases simulated (CS0, CS1, CS2) toward one of the favorable collateral development cases (CS3, CS4) as the LCN vascular resistance decreases due to vasodilation. The capacity for additional collateral recruitment through vasodilation is largely dependent on individual factors such as age or history of chronic hypertension (Li et al. 2024). From the results of our study, we can also see the importance of the primary cerebral collaterals (i.e., PComAs and AComA) in a complete CoW to maintaining balance between the left and right cerebral arteries. In Fig. 7a, we observed that, regardless of collateral development level, the flow distribution remained relatively balanced between the patent vessels contributing to the left and right brain hemispheres despite there being an imbalance in the flow between the MCAs due to the LMCA occlusion. This is an important regulatory feature of the cerebral vasculature that helps maintain sufficient flow to the entire brain volume.

**Fig. 8:**
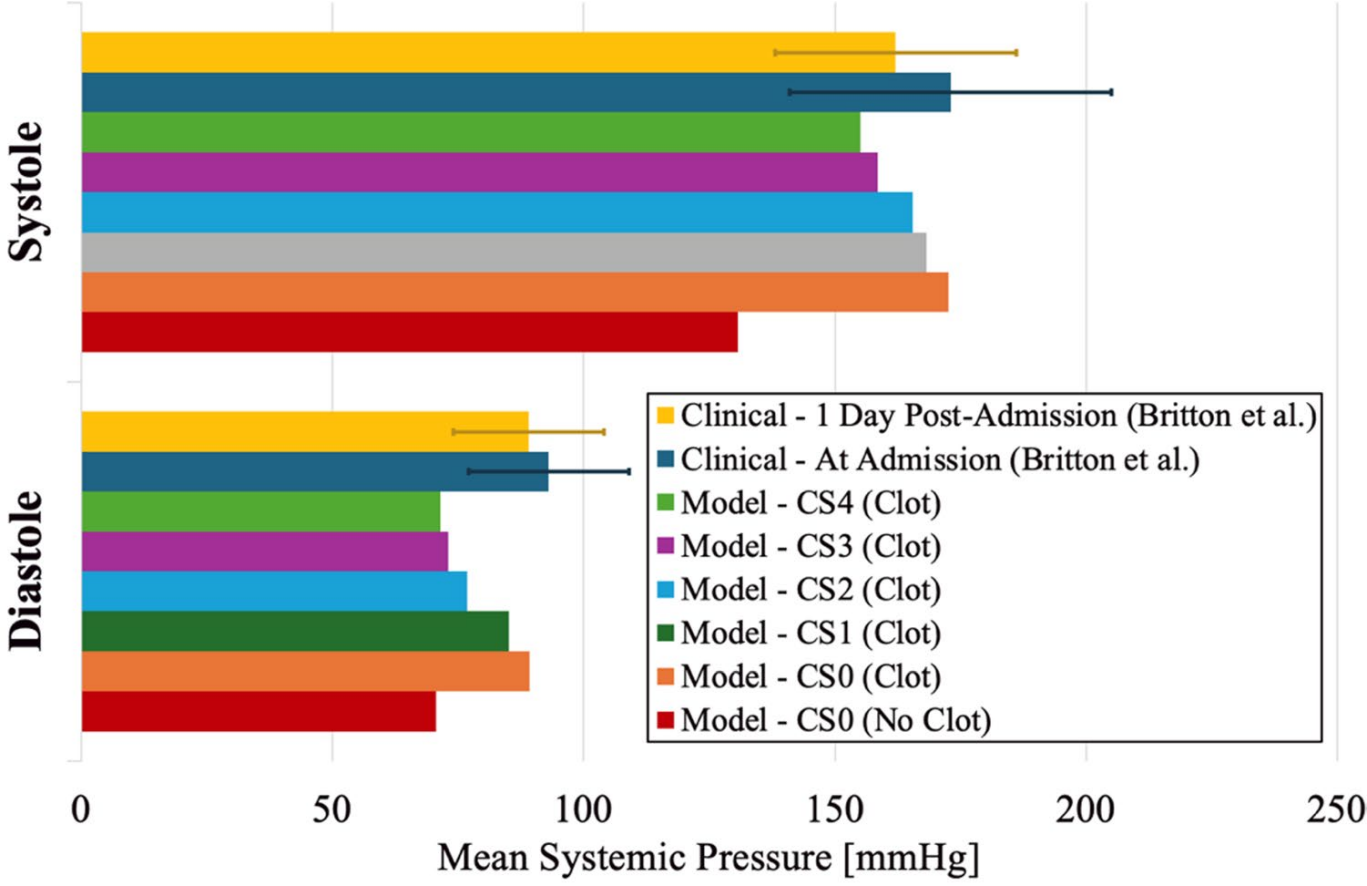
Bar graph comparison of computational predictions of sSBP and sDBP for each computational case compared to clinically measured systemic pressures in patients with embolic stroke at two time points: hospital admission and one day after admission (Britton et al. 1986)

AIS patients present to the hospital with elevated systemic blood pressure, and this phenomenon was captured in our model. The mechanisms behind this elevated BP are not well-described but are likely tied to the increase in total cerebral vascular resistance following occlusion and the body attempting to maintain sufficient total CBF. A previous clinical study showed that patients admitted with embolic AIS had elevated sSBP and sDBP on admission, measuring an average of 173 and 93 mmHg, respectively (Britton et al. 1986). They also observed the elevated systolic and diastolic blood pressures decreased with time following admission, averaging 162 and 84 mmHg, respectively one day post-admission. These clinical observations align well with the systemic pressures predicted in the computational model (Fig. 8). The higher clinical admission sSBP was closest to the sSBP of the absent (CS0) and poor (CS1) collateral cases, which were 173 and 168 mmHg, respectively. In contrast, the clinical one day post-admission sSBP was closer to that of the more highly developed collateral cases (CS2 and CS3), which were 165 and 159 mmHg, respectively. These results indicate that long-term collateral recruitment over the course of hours to days after occlusion may factor into the clinically observed systemic sSBP reduction in patients as the cerebral autoregulatory network attempts to return to normal physiological hemodynamic conditions. The decrease in the sDBP observed clinically the day following admission was not as severe as the decrease in sSBP. Both the at admission and 1 day post-admission sDBP values matched best with the absent and poor collateral development cases (CS0 and CS1), as shown in Fig. 8, which indicates that collateral recruitment does not reduce sDBP pressure as much as the model predicts. The deviation between the model and clinical observations here may be a result of the model failing to capture the altered vessel compliance during the vasodilation phase of LCN recruitment that can reduce the pulse pressure.

### Hemodynamics and biomechanical forces related to IVT and EVT therapies

The pressure near the proximal and distal face of the occluding clot was extracted because the pressure drop across the clot plays a large role in both IVT and EVT therapies for vessel recanalization. In the case of IVT, the pressure drop has two competing effects that can alter thrombolytic susceptibility of the clot. First, the pressure drop is directly related to the pressure gradient that drives interstitial flow through the porous clot scaffold. Increased interstitial blood flow due to higher pressure gradients can mediate more efficient advective transport of thrombolytic agents to the inner region of the clot (Tratar et al. 2004). Thus, from strictly a fluid dynamics perspective, increased pressure differences across the clot can lead to faster dissolution by exposing a greater amount of fibrin in the clot to the thrombolytics. However, increased pressure differences can produce contradictory effects due to higher compaction pressures on the clot. Clot compaction alters the microstructure by packing clot constituents together and reducing porosity, which reduces the clot permeability and susceptibility to thrombolytics (Dwivedi et al. 2021; Thalerová et al. 2024). The pressure drop is also an important factor to EVT because the distal compaction forces on the clot impact the force required to pull the clot out of the vessel (Froehler and Good 2025). This principle is applicable to all forms of EVT, including both aspiration and stent retriever thrombectomies. A previous computational study evaluated pressure drops over an occlusive clot in the MCA in a one-dimensional steady flow model and found the drop ranged from 60 to 85 mmHg, depending on the LCN development level (Padmos et al. 2021). Our findings are consistent with their results in terms of both the pressure drop magnitude and the trend of lower pressure drop magnitudes in cases with more favorable collateral development, since the pressure drop across the occlusive clot in our study changed from 118 mmHg in the CS0 case to 40 mmHg in the CS4 case.

As mentioned previously, the present study found that lower levels of collateralization led to greater increases in sSBP, sDBP, and systemic MAP and lower pressures in the distal LMCA following occlusion, which resulted in greater pressure drops across the clot. The net distal compaction force can be calculated by assuming the clot is static and the only forces acting it are due to the proximal and distal pressures, balanced by the external and internal frictional forces arising from interactions with the vessel wall and clot components during compaction, respectively (Froehler and Good 2025). The values for net distal compaction forces on the occlusive clot can be compared to the forces generated by aspiration catheters, which are the current clinical gold standard for EVT, to evaluate the potential for clot removal and recanalization success. Cases where the vacuum forces generated at the aspiration catheter tip do not surpass the distal compaction force would indicate EVT failure. The static pressure force generated by an aspiration catheter tip at the proximal clot surface (${F}_{cath}$) can be estimated using **Eq. 8.**

8 $${F}_{cath}={P}_{v}\left(\frac{\pi }{4}{D}_{cath}^{2}\right)$$

where ${P}_{v}$ is the vacuum pressure generated by the aspiration thrombectomy system and ${D}_{cath}$ is the internal lumen diameter of the aspiration catheter. This equation assumes the catheter tip has a circular cross section and applies a constant vacuum pressure, both of which are valid assumptions for most current aspiration thrombectomy systems, apart from the few that apply cyclic aspiration (Patki et al. 2023, 2024; Wei et al. 2024).

To overcome the larger pressure differences across clots in patients with a poor LCN development, aspiration systems that produce larger vacuum pressures or larger aspiration catheters must be used. Yaeger et al. (2020) reported the average tip forces generated by 8 different commercially available aspiration catheters with varying internal diameters when attached to the Penumbra Jet Engine aspiration system. The forces ranged from 56.2 to 315.0 mN and experimental data showed a linear relationship between tip force generated and catheter internal diameter. The maximum distal compaction forces occurring during systole calculated from our computational study ranged from 82.54 to 172.70 mN, with higher forces occurring in cases with poorer collateral development. Poor collateral development was also associated with higher pressure drops across the clot during diastole that produced elevated minimum compaction forces experienced by the occlusive clot. The minimum compaction forces ranged from 28.38 mN with favorable collateral development (CS4) to 89.24 mN with absent collaterals (CS0). These data show that even when neglecting additional frictional forces between the clot and vessel wall during removal, which themselves can be on the order of tens to hundreds of mN (Romero et al. 2010; Chueh et al. 2012), certain aspiration catheters with smaller diameters may not be able to generate sufficient forces for clot removal, particularly in cases where there is poor collateralization.

The results of this study demonstrate the utility of considering collateral vessel development level and distal clot compaction forces during EVT surgical planning. Understanding the minimum force required to overcome the compaction forces alone is important when choosing the aspiration catheter and vacuum pump system for two reasons. First, avoiding an initial choice of a catheter–pump combination that is incapable of overcoming the distal clot compaction force will help reduce the average number of passes required and the overall time to recanalization. Second, the surgeon can choose a catheter–pump combination that does not drastically exceed what is required to overcome the distal clot compaction force. Larger aspiration catheters and higher vacuum pressures are more likely to lead to adverse events such as endothelial damage, intracranial bleeding, or vessel collapse during surgery (Pérez-García et al. 2021; Liu et al. 2022a, b; Schartz et al. 2023; Patki et al. 2024). Certain large catheters may also not be a viable option depending on the size and tortuosity of the patient vasculature leading to the occlusion site. Therefore, consideration of the relationship between distal clot compaction force and the level of LCN development can help surgeons find an optimal EVT system that balances efficiency and safety based on patient-specific demands. For example, when assessing the extreme CS0 anatomical case with no collateral vessels present that produced the largest distal compaction force of 172.70 mN, the minimum internal catheter diameter required to overcome the distal compaction force would be 0.061″ when using the Penumbra Jet Engine aspiration system. Whereas for the other extreme case of highly developed collaterals (CS4), the maximum distal compaction force is only 82.54 mN, which corresponds to a minimum catheter internal diameter of 0.043″. These results emphasize the importance of being able to accurately assess collateralization to optimize patient-specific treatment plans.

Previous computational studies of aspiration thrombectomy showed that clot length and pressure drop across a clot affect the required aspiration pressure to cause detachment from the vessel wall to initiate clot removal and the detachment behavior (Patki et al. 2023; Monclova et al. 2025). AIS cases with greater distal compaction forces resulting from poor collateral development would likely experience clots being pushed further distally into the vessel to reach equilibrium state compared to cases with well-developed collaterals. Assuming incompressibility, a clot being pushed distally would result in an increase in total clot length as it deforms to match the shape of the tapering vessel while maintaining its volume. Clot lengthening increases the minimum aspiration pressure required to detach the clot from the wall (Monclova et al. 2025). Additionally, the increased length alters the detachment point from the wall and the internal stresses experience by the clot, which can make it more susceptible to fracture during removal (Patki et al. 2023). Both these effects can reduce the likelihood of recanalization success and lead to less favorable patient outcomes, thus pointing to an additional mechanism by which level of collateral development may influence clinical results for EVT.

### Future directions and limitations

The current study has demonstrated that we can incorporate the simplified collateral network model into a three-dimensional, realistic, patient-specific vasculature to alter the hemodynamics in a controlled manner to reflect physiological hemodynamic changes during LVO accurately. In future studies, combining this CFD model with a computational EVT model, similar to the ones mentioned previously, or an IVT model, will aid in developing a comprehensive approach for predicting responses to reperfusion therapies accurately based on the surrounding hemodynamic environment derived from patient-specific parameters, including vessel anatomy, systemic pressure, flow distribution, and level of collateral development. Although a one-dimensional model may have been sufficient to provide similar results within the scope of the present study while reducing computational cost, one-dimensional modeling would be insufficient to model the complex transport, forces, and solid-body deformation accurately affecting the system during reperfusion therapies. Thus, this study lays down a critical foundation for future work related to computational modeling of AIS and treatment.

Although the computational model presented in this study has made improvements over previous models, there are limitations. First, the governing fluids equation assumed Newtonian behavior of the blood. This assumption is valid for most of the modeled anatomy where shear rates are high. However, there are certain regions where this assumption may be invalid. For instance, the Newtonian model may not accurately represent blood viscosity in the low shear regions near bifurcations and branches. Newtonian blood behavior can also not be assumed in the small vessels that comprise much of the true LCN. Although the Newtonian assumption is valid for the consolidated single collateral model used, this model will fail to capture hemodynamic changes stemming from the non-Newtonian behavior in the small collateral vasculature, thus limiting the scope of this model to the large vessel CoW hemodynamics and precluding conclusions from being drawn regarding region-specific CBF and perfusion.

Second, the current model assumes that the flow distribution between the left and right ICA, as well as the total CBF remains constant following LVO of the proximal MCA. A previous clinical study of cerebral blood flow distributions in a complete CoW and through patients with missing or hypoplastic A1 ACA showed that the elevated resistance on the side of the pathological vessel caused a 25% inflow reduction in the ipsilateral ICA and a compensatory 25% inflow increase through the contralateral ICA (Zarrinkoob et al. 2015). Thus, there may be a shift in flow distribution toward the contralateral hemisphere that we are not currently capturing based on the constant distribution assumption. To account for this in future studies, the anatomical model could either be extended down to the origins of the LICA and RICA along the aorta to capture the flow redistribution due to the altered hemodynamic resistances within the cerebral vasculature or the inlet boundary conditions for velocity could be adjusted to match clinically observed changes.

The current study focuses on only one anatomical model that is a complete CoW with average pressure and flow distributions. This was chosen because it is the best representative example to be applicable for many AIS cases broadly because a complete CoW is the most common anatomical presentation, which makes the results more generalizable to the population (Alpers 1959). However, the CoW anatomy can be highly variable between patients. There are many incomplete CoW variations, including cases with missing or narrowed AComA and PComA (Hindenes et al. 2020). The present study uses an anatomical model with patent and intact communicating arteries. The results demonstrate that both communicating arteries play a critical role in the primary collateral pathways affecting hemodynamic shifts during LVO. Thus, it can be hypothesized that the hemodynamic changes following LVO in anatomical variants of the CoW where these vessels are not present may be significantly different than what the current model predicts, potentially leading to higher systemic pressure increases and reduced compensation capacity for flow distal to the occlusion. Additional studies should be completed to assess the effect of CoW anatomical variations on hemodynamic responses during LVO.

Finally, the consolidated single vessel LCN model does not capture the complex physiological response of arteries and arterioles within the LCN. Dynamic compensatory responses of the cerebral autoregulatory system, such as acute vasodilation of the LCN vessels during LVO, will result in time-dependent hemodynamic changes in the CoW (Castro et al. 2018). This complex physiology is not captured by the static anatomical model used in the present study, which models the LCN as having a constant hemodynamic resistance that does not adapt based on the cerebral hemodynamics, like in vivo. The purpose of this study was to assess acute hemodynamic responses in the CoW and the resultant forces experienced by the occlusive clot. The vasodilation and vasoconstriction are not expected to play as significant of a role within the short time window assessed immediately following occlusion compared to the existing collateral development level. Therefore, the exclusion of this phenomenon from the model does not diminish the results presented in the study but strictly limits the temporal scope to the acute phase following occlusion. Extending this model to include a time-dependent resistance element into the simulated collateral network, similar to previous autoregulatory models (Kim 2007; Alzaidi 2009; Padmos et al. 2021; Tong et al. 2021), could help uncover long-term changes that may occur over the hours from stroke onset to treatment using a single anatomical model with tunable resistance that receives feedback based on hemodynamic changes and demand.

## Conclusions

In conclusion, the results of our computational study evaluating flow in realistic three-dimensional cerebral anatomical models have provided critical insights into the complex hemodynamic changes in the CoW following MCA occlusion, which have been used to clarify the mechanisms that underlie the established correlations between individual collateral development level and clinical outcomes of stroke patients. From our study, we can identify two separate mechanisms that are important to predicting patient outcomes and effective treatment strategies: (1) Lower pressure gradients across the clot in anatomies with a favorable LCN development reduce clot compaction pressures and leave the occlusive clot more susceptible to IVT and EVT. (2) Better collateral recruitment capacity in anatomies with well-developed LCNs is able to compensate for a greater amount of the occluded vessel flow loss to maintain sufficient blood perfusion to the affected brain tissue and reduce the infarct volume growth rate.

## Figures and Tables

**Fig. 9:**
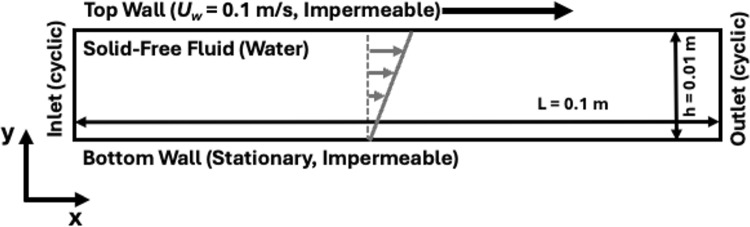
Computational model setup schematic illustrating the geometry and boundary conditions for the canonical Couette flow case of the verification study

**Fig. 10:**
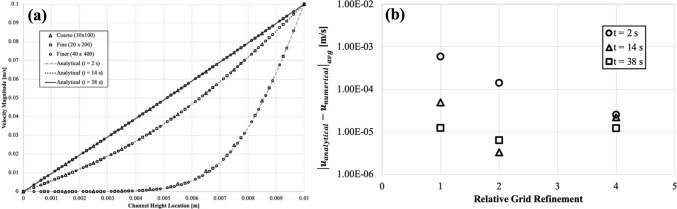
(**a**) Velocity profiles across the height of the channel for three time points show agreement between the numerical and analytical solutions, and (**b**) average absolute velocity error plots at each location along the channel height shows convergence of the numerical solution to the analytical solution

**Fig. 11:**
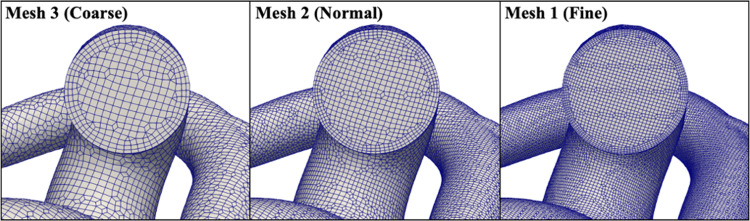
Representative images of the three meshes with different refinement levels used in the grid independence study, as viewed from the LICA inlet

## Data Availability

Data sharing is not applicable to this article as no datasets were generated or analyzed during the current study.

## Appendix A: Code verification study

The computational case for the verification study was set up to match the canonical unsteady 2D planar Couette flow of an incompressible Newtonian fluid in a solid-free domain using the boundary conditions defined in Fig. 9. The initial conditions defined the velocity to be equal to zero everywhere except along the top wall boundary. The fluid kinematic viscosity and density were set to 1.1 × 10^–6^ m^2^/s and 1000 kg/m^3^, respectively. Second-order linear spatial differencing schemes and a second-order implicit time differencing scheme were used to numerically solve the modified Navier–Stokes equation, and the computational time step was modified to maintain a Courant number < 0.2. The numerical convergence criteria for velocity and pressure were 10^–6^ and 10^–5^, respectively.

The initial Darcy–Brinkman modified Navier–Stokes equation governing the fluid dynamics defined in the main text (Eq. 2) was reduced using the simplifying assumptions of the canonical Couette flow case. The simplifying assumptions applied to get the final reduced form equation (Eq. 6) included 2D unsteady (${{\boldsymbol{u}}}_{{\boldsymbol{f}}}={{\boldsymbol{u}}}_{{\boldsymbol{f}}}\left(\mathrm{y},\mathrm{t}\right)$) flow, no driving pressure gradient ($\nabla p$ = 0), a Newtonian and incompressible fluid (${\nu }_{f}, {\rho }_{f}$ = constant), and solid-free flow ($\Phi$ = 1).

6 $$\frac{\partial {{\boldsymbol{u}}}_{{\boldsymbol{f}}}}{\partial t}+\left({{\boldsymbol{u}}}_{{\boldsymbol{f}}}\cdot \nabla \right){{\boldsymbol{u}}}_{{\boldsymbol{f}}}={\nu }_{f}{\nabla }^{2}\left({{\boldsymbol{u}}}_{{\boldsymbol{f}}}\right)$$

The analytical solution for the equation above has been defined previously (Eq. 7).

7 $${u}_{f,x}\left(y,t\right)={U}_{w}\frac{y}{h}-\frac{2{U}_{w}}{\pi }\sum_{n=1}^{\infty }\frac{1}{n}{e}^{-{n}^{2}{\pi }^{2}\frac{{\nu }_{f}t}{{h}^{2}}}\mathrm{sin}[n\pi \left(1-\frac{y}{h}\right)]$$

The analytical and numerical solutions for three time points (t = 2, 14, 38 s) on three different computational grids of increasing refinement were computed. The results verified our numerical solver by confirming that the calculated numerical velocity profiles matched those of the analytical solution for all y-locations at all three time points (Fig. 10a) and the numerical solution converged to the analytical solution with increasing grid refinement (Fig. 10b). The final absolute velocity error was approximately 10^–5^, which matched the order of magnitude of the numerical solver convergence criteria.

## Appendix B: Grid independence study

Identical simulations of flow through the CS4 anatomical model with an M1-LMCA occlusive clot were performed on three different meshes of increasing refinement (Fig. 11). Two outputs of interest related to this study, mean distal clot compaction force (*f*) and ipsilateral collateral flow rate (*g*), were extracted and compared across the three mesh refinement cases to assess convergence based on the Roache grid convergence index (GCI) (Table

**Table 7:** Summary of the properties of the three meshes in the grid refinement study and the outputs of interest

Computational Mesh	Total Number of Mesh Cells	Characteristic Cell Size (${\boldsymbol{h}}$)	Mean Clot Force (${\boldsymbol{f}}$)	Ipsilateral Mean Flow (${\boldsymbol{g}}$)
Mesh 3	223,002	0.3 mm	48.06 mN	93.88 mL/min
Mesh 2	634,941	0.2 mm	47.81 mN	98.21 mL/min
Mesh 1	2,133,715	0.133 mm	47.75 mN	98.82 mL/min

7). The cases were designed so that all parameters were the same for all three grids and matched those of the computational studies presented here. Both the numerical uncertainty and asymptotic convergence criteria calculations are detailed in Table

**Table 8:** Calculations of the Roache GCI uncertainty and asymptotic grid convergence using both outputs of interest

Roache GCI & Discretization Uncertainty Calculations	
Mean Clot Force ($f$)	Ipsilateral Mean Collateral Flow Rate ($g$)
${{\boldsymbol{r}}}_{{\boldsymbol{f}}}= \frac{{{\boldsymbol{h}}}_{2}}{{{\boldsymbol{h}}}_{1}}=\frac{{{\boldsymbol{h}}}_{3}}{{{\boldsymbol{h}}}_{2}}=1.5$ ${{\boldsymbol{\varepsilon}}}_{{\boldsymbol{f}}}=\frac{{{\boldsymbol{f}}}_{2}-{{\boldsymbol{f}}}_{1}}{{{\boldsymbol{f}}}_{1}}=0.001257$ ${{\boldsymbol{p}}}_{{\boldsymbol{f}}}=\frac{\mathbf{ln}\left(\frac{{{\boldsymbol{f}}}_{3}-{{\boldsymbol{f}}}_{2}}{{{\boldsymbol{f}}}_{2}-{{\boldsymbol{f}}}_{1}}\right)}{\mathbf{ln}\left({\boldsymbol{r}}\right)}=3.5197$ ${{\boldsymbol{G}}{\boldsymbol{C}}{\boldsymbol{I}}}_{{\boldsymbol{f}}}=100\%\bullet \frac{1.25\left|{{\boldsymbol{\varepsilon}}}_{{\boldsymbol{f}}}\right|{{{\boldsymbol{r}}}_{{\boldsymbol{f}}}}^{{{\boldsymbol{p}}}_{{\boldsymbol{f}}}}}{\left({{{\boldsymbol{r}}}_{{\boldsymbol{f}}}}^{{{\boldsymbol{p}}}_{{\boldsymbol{f}}}}-1\right)}=0.21\%$	${r}_{g}= \frac{{h}_{2}}{{h}_{1}}=\frac{{h}_{3}}{{h}_{2}}=1.5$ ${\varepsilon }_{g}=\frac{{g}_{2}-{g}_{1}}{{g}_{1}}=-0.006173$ ${p}_{g}=\frac{\mathrm{ln}\left(\frac{{g}_{3}-{g}_{2}}{{g}_{2}-{g}_{1}}\right)}{\mathrm{ln}\left(r\right)}=4.8336$ ${GCI}_{g}=100\%\bullet \frac{1.25\left|{\varepsilon }_{g}\right|{{r}_{g}}^{{p}_{g}}}{\left({{r}_{g}}^{{p}_{g}}-1\right)}=0.90\%$

Asymptotic Grid Convergence Confirmation	
Mean Clot Force ($f$)	Ipsilateral Mean Collateral Flow Rate ($g$)
${{\boldsymbol{G}}{\boldsymbol{C}}{\boldsymbol{I}}}_{{\boldsymbol{f}}12}=100\%\bullet \frac{1.25\left|{{\boldsymbol{\varepsilon}}}_{{\boldsymbol{f}}}\right|}{\left({{{\boldsymbol{r}}}_{{\boldsymbol{f}}}}^{{{\boldsymbol{p}}}_{{\boldsymbol{f}}}}-1\right)}=0.0496\%$ ${{\boldsymbol{G}}{\boldsymbol{C}}{\boldsymbol{I}}}_{{\boldsymbol{f}}23}=100\%\bullet \frac{1.25\left|{{\boldsymbol{\varepsilon}}}_{{\boldsymbol{f}}}\right|}{\left({{{\boldsymbol{r}}}_{{\boldsymbol{f}}}}^{{{\boldsymbol{p}}}_{{\boldsymbol{f}}}}-1\right)}=0.2064\%$ *Asymptotic convergence criteria:* $\frac{{{\boldsymbol{G}}{\boldsymbol{C}}{\boldsymbol{I}}}_{{\boldsymbol{f}}23}}{{{\boldsymbol{G}}{\boldsymbol{C}}{\boldsymbol{I}}}_{{\boldsymbol{f}}12}}\approx {{{\boldsymbol{r}}}_{{\boldsymbol{f}}}}^{{{\boldsymbol{p}}}_{{\boldsymbol{f}}}}$ $4.1614\approx 4.1667$	${GCI}_{g12}=100\%\bullet \frac{1.25\left|{\varepsilon }_{g}\right|}{\left({{r}_{g}}^{{p}_{g}}-1\right)}=0.1265\%$ ${GCI}_{g23}=100\%\bullet \frac{1.25\left|{\varepsilon }_{g}\right|}{\left({{r}_{g}}^{{p}_{g}}-1\right)}=0.9037\%$ *Asymptotic convergence criteria:* $\frac{{GCI}_{g23}}{{GCI}_{g12}}\approx {{r}_{g}}^{{p}_{g}}$ $7.1439\approx 7.0984$

^8^ based on methods detailed in (Roache 1994).

## Abbreviations

EVT: Endovascular Thrombectomy
IVT: Intravenous Thrombolytic Therapy
CBF: Cerebral Blood Flow
MAQ: Mean Arterial Flow Rate
MAP: Mean Arterial Pressure
sSBP: Systemic Systolic Blood Pressure
sDBP: Systemic Diastolic Blood Pressure
AIS: Acute Ischemic Stroke
LVO: Large Vessel Occlusion
LCN: Leptomeningeal Collateral Network
CFD: Computational Fluid Dynamics
CoW: Circle of Willis
CTA: Computed Tomography Angiography
MRI: Magnetic Resonance Imaging
PCMRI: Phase Contrast Magnetic Resonance Imaging
ACA: Anterior Cerebral Artery
AComA: Anterior Communicating Artery
ICA: Internal Carotid Artery
MCA: Middle Cerebral Artery
PCA: Posterior Cerebral Artery
PComA: Posterior Communicating Artery
SCA: Superior Cerebellar Artery
BA: Basilar Artery
